# Supplementary material for: Rubisco is evolving for improved catalytic efficiency and CO2 assimilation in plants
Source: Proc Natl Acad Sci U S A. 2024 Mar 5;121(11):e2321050121. doi: 10.1073/pnas.2321050121 (PMC10945770; doi:10.1073/pnas.2321050121)
Supplement: Supplementary file 1 — Appendix 01 (PDF) [file pnas.2321050121.sapp.pdf]

Supplemental Results

Form I rubisco ancillary chaperones exhibit a variable rate of molecular evolution

The molecular evolution of rubisco interacting partners were evaluated following the same method used to compute the percentile rank rate of evolution in *rbcL*/RbcL and *rbcS*/RbcS. These interacting partners included components involved in holoenzyme metabolic regulation (RUBISCO ACTIVASE (Rca)) as well as in holoenzyme folding and assembly (CHAPERONIN 10 (Cpn10), CHAPERONIN 20 (Cpn20), CHAPERONIN 60 (Cpn60), RBCX (RbcX), RUBISCO ACCUMULATION FACTOR 1 (Raf1), RUBISCO ASSEMBLY FACTOR 2 (Raf2)). This analysis revealed that the percentile rate of molecular evolution in rubisco chaperones was highly variable across all taxonomic groups, with no consistent pattern emerging in the relative extent of these genes compared to that experienced by the cohort of all other genes encoded in each species genome (Figure S3A and S3B and table S11). Thus, similar to the case in the rubisco small subunit, rubisco chaperones do not experience the ubiquitous slow pace of molecular evolution observed in the large subunit of the holoenzyme.

Correlations in rubisco molecular and kinetic evolution are robust to biases in species sampling and model of sequence evolution

In order to evaluate the robustness of our integrated molecular and kinetic analysis, we conducted a systematic and comprehensive examination of possible methodological biases associated with our method to determine whether these may have impacted the results presented. Specifically, two such potential methodological issues were identified and include biases arising from incomplete species sampling in the rubisco kinetic dataset as well as biases arising from uncertainties or errors in the underlying phylogenetic gene tree that is used as the basis of the analysis. To address each of these in turn, a supporting set of additional analyses were designed. The results generated from these analyses were compared to those generated from the original analysis to assess the reliability of the presented conclusions.

First, we repeated the analysis of rubisco molecular and kinetic evolution but including only the minimum subset of species that captured most phylogenetic diversity across the species in the full analysis (see Methods, Figure S4). This additional analysis thus addresses any potential

methodological artefacts caused by biases associated with incomplete species sampling (including overrepresentation of certain clades on the angiosperm phylogenetic tree of life).

Next, we repeated the analysis of rubisco molecular and kinetic evolution but using RbcL phylogenetic gene trees inferred from the same sequence alignment but using alternate models of sequence evolution. This analysis thus addresses any potential methodological artefacts associated with sources of error in the underlying phylogenetic tree caused by assuming a particular model of sequence evolution.

Analogous conclusions were generated from the results of each of these analyses (table S12 and S13) to those presented in the original investigation (Figure 4B). Thus, we confirm that the results presented in our study are robust and are not an artefact caused by systematic biases in species sampling or caused by errors in phylogenetic reconstruction.

Supplemental Materials and Methods

Stratified sampling of rbcS sequences and phylogenetic tree inference

To account for potential biases in our analysis caused by some species exhibiting multiple copies of *rbcS*, random stratified sampling of the non-gapped *rbcS*/RbcS sequence alignments was conducted by species using 1,000 replicates with replacement. This process resulted in the generation of 1,000 unique *rbcS*/RbcS alignments for each taxonomic group, whereby each of these respective alignments contain only a single randomly selected copy of the *rbcS*/RbcS per species. In turn, each of these alignments were subject to data processing and phylogenetic tree inference using IQ-TREE[1] following the method previously described.

Genomes and gene models

Complete sets of representative gene models for as many species in the rubisco sequence dataset as possible were acquired from either NCBI (https://www.ncbi.nlm.nih.gov/) or Phytozome V13[2]. Where more than one such gene model resource was available for a given species, the most recent assembly version was chosen. In this way, complete sets of representative gene models were acquired for a total of 32 of the bacteria species, 27 of the land plant species, 8 of the SAR species,

6 of the red algae species and 4 of the green algae species analysed in the present study, respectively (table S1 and S7).

Predicted gene model sets were filtered to remove sequences with internal in-frame stop codons. Gene model sets were also filtered to keep only the longest gene model variant per gene. Moreover, owing to a lack of data availability of publicly available chloroplast or mitochondrial genomes for the eukaryotic species in the present analysis, and as organellar genomes contain fewer than 1% of genes encoded in the corresponding nuclear genome, only gene sequences encoded by the nuclear genomes of species in the land plant, green algae, red algae and SAR taxonomic groups were taken forward for analysis. Finally, after the above quality control checks were completed, a corresponding proteome was generated from each species gene model set by *in silico* translation of the respective coding sequences.

Characterization of the set of enzymatic gene and protein sequences within orthogroups

The set of all genes within each species proteome that encode enzymes was determined using the DeepEC[3] deep learning-based classifier algorithm. For this purpose, enzymes were defined as those protein sequences that could be assigned at least a partial enzyme commission (EC) number (i.e., at minimum, a single digit EC top-level code). On average 42.2% of all genes in the analysis encoded enzymes. A detailed breakdown of the metrics of enzyme ortholog pairwise comparisons for each species comparison can be found in Supplemental File 4.

Identification and classification of Calvin-Benson-Bassham cycle enzyme isoforms in land plants

The set of genes which encode Calvin-Benson-Bassham cycle enzymes was first resolved in the model plant species *Arabidopsis thaliana*. To achieve this, the complete gene families to which each Calvin-Benson-Bassham cycle enzyme in *A. thaliana* belongs was determined based on available data in The Arabidopsis Information Resource (TAIR) database (http://arabidopsis.org)[4,5]. Following this, the photosynthetic isoforms in these gene families which are active in the Calvin-Benson-Bassham cycle in the chloroplast stroma were then identified based on several lines of evidence. 1) A high protein abundance based on whole-organism integrated protein abundance data obtained

from the Protein Abundance Database (https://pax-db.org/) dataset 3702/323. 2) Leaf mRNA expression based on tissue-specific RNA sequencing data obtained from both the Arabidopsis eFP Browser V2.0 (http://bar.utoronto.ca/efp2/Arabidopsis/Arabidopsis_eFPBrowser2.html) and the EMBL-EBI (https://www.ebi.ac.uk/) dataset E-GEOD-53197. 3) Chloroplast-targeted protein subcellular localisation as predicted using both TargetP V2.0[6,7] and Predotar V1.04[8]. 4) Gene orthology as inferred from trees generated for each Calvin-Benson-Bassham cycle gene family using IQ-TREE[1]. The resulting set of photosynthetic isoforms encoding each Calvin-Benson-Bassham cycle enzyme in *A. thaliana* inferred from this multi-faceted analytical pipeline can be found in table S8.

The set of genes which encode the photosynthetic isoforms of Calvin Bensen Bassham cycle enzymes in all other 26 land plant species (apart from *A. thaliana*) for which genome sequence data was available in this study were then determined by orthology using data from the orthogroup inference analysis previously performed. Each group of orthologous protein sequences determined to encode a given Calvin-Benson-Bassham cycle enzyme across all species were aligned using the MAFFT L-INS-i algorithm[9], and corresponding nucleotide coding sequence alignments were generated using PAL2NAL[10]. Multiple sequence alignments were subject to the same data filtering and quality control criteria previously described to remove partial or incomplete sequences and subsequently delete any column positions which contain gaps. Finally, bootstrapped maximum likelihood phylogenetic trees were inferred by IQ-TREE[1] following the method previously described. A similar analysis of Calvin-Bensen-Bassham cycle enzymes in other taxonomic groups was not able to be performed owing to a lack of the required data described here to determine the photosynthetic gene isoforms in these species.

Quantification of the relative extent of rubisco molecular evolution compared to all other Calvin-Benson-Bassham cycle enzymes in land plants

To determine the difference in evolutionary rate between rubisco and all other Calvin-Benson-Bassham cycle enzymes, the extent of molecular evolution measured between all other Calvin-Benson-Bassham cycle gene and protein orthologous sequences for each species pair (including *rbcS*/RbcS) were expressed as a percentage ratio of that measured in the corresponding *rbcL*/RbcL

sequence. In cases where multiple percentage ratios are calculated for a given Calvin-Bensen-Bassham component in a given species pair (due to gene duplications, or due to a single species gene assembly matching multiple sub-species in the rubisco sequence dataset) the mean value was taken. The processed data generated from this analysis can be found in Supplemental File 5.

Quantification of the percentile extent of rubisco chaperone molecular evolution within each taxonomic group

To evaluate the percentile rate of molecular evolution in the known chaperones of Form I rubisco in the context of all other genes in each taxonomic group, the exact same method was followed as previously described for *rbcL*/RbcL and *rbcS*/RbcS though the subject of the analysis was respectively altered. Here, for this investigation the putative set of genes which encode each Form I ancillary chaperone involved in holoenzyme metabolic regulation (RUBISCO ACTIVASE (Rca)) and in holoenzyme folding and assembly (BUNDLE SHEATH DEFECTIVE 2 (BSD2), CHAPERONIN 10 (Cpn10), CHAPERONIN 20 (Cpn20), CHAPERONIN 60 (Cpn60), RBCX (RbcX), RUBISCO ACCUMULATION FACTOR 1 (Raf1), RUBISCO ASSEMBLY FACTOR 2 (Raf2)) were first resolved in the model plant species *A. thaliana*. This was achieved using a previously published dataset[11] supplemented by information available in the TAIR database (http://arabidopsis.org)[4,5]. The resulting set of *Arabidopsis* chaperone genes thus identified can be found in table S9. Following this step, the corresponding set of genes encoding rubisco chaperones in all other species for which a complete gene assembly could be obtained were inferred using data from a separate OrthoFinder run performed with identical settings as previously described, but based on the translated proteomes of all organisms across all taxonomic groups together.

After the cohort of Form I rubisco chaperone genes were identified in all species, the percentile rates of nucleotide and protein evolution in these genes were calculated between each pairwise combination of species relative to all other pairs of orthologous sequences using the identical measurements previously generated from the analysis of *rbcL*/RbcL and *rbcS*/RbcS. In this way, analysis of some chaperones were omitted in certain taxonomic groups owing to the data quality and filtering steps that were performed as previously described. In cases where multiple percentiles are calculated for a chaperone in a given species pair (due to gene duplications, or due to a single

species gene assembly matching multiple sub-species in the rubisco sequence dataset) the mean percentile was taken as previously described. The processed data quantifying the relative percentile extent of molecular evolution in rubisco chaperones in the context of all other genes and proteins can be found in Supplemental File 5 and Supplemental File 6.

Accounting for potential errors in the integrated analysis of rubisco molecular and kinetic evolution associated with species sampling and model of sequence evolution

To control for potential errors associated with species sampling and overrepresentation of certain groups in the integrated molecular and kinetic investigation of rubisco evolution, an identical analysis was performed but including only the minimal subset of studied species which captured the majority of phylogenetic diversity (PD) in the kinetic dataset. For this purpose, the minimal subset of phylogenetically diverse studied species was identified by employing the Phylogenetic Diversity Analyzer V1.0.3 software[12] with the 'greedy' algorithm. Here, PD is defined as the total tree length (i.e., the combined sum of all internal and terminal branch lengths) of the pruned phylogeny comprising a selected subset of sampled species. In brief, the unrooted RbcL phylogenetic tree of the 93 C_3 species was subject to systematic interrogation to identify the optimal combination of species at each iterative tree size (n = 2 – 93 species) which maximizes the PD score. Based on the results of this analysis, it was observed that PD plateaued at a threshold of 50 species (accounting for 54.8% of the total 93 C_3 species in the kinetic dataset) (Figure S4). The optimal composition of species at this respective threshold used for the basis of subsequent analyses included 31 dicotyledonous individuals and 19 monocotyledonous individuals, and are listed in table S10.

To control for potential artefacts associated with errors or uncertainties in phylogenetic tree inference in the integrated molecular and kinetic investigation of rubisco evolution, an identical analysis was performed using the complete set of species in the kinetic dataset but based on analogous gene trees. Specifically, these gene trees were generated using the same alignment of sequences and following the exact same method as previously described, but based on alternate best-fitting models of sequence evolution (LG+I+G4 and JTTDCMut+I+G4, respectively).

As the results of both of the above supplementary analyses based on the minimal subset of phylogenetically diverse species and based on alternate gene trees were identical to those generated from the original analysis, the conclusions in the present study were demonstrated to be valid and robust. Thus, the analyses presented are not an artefact caused by either systematic biases in species sampling or by errors in phylogenetic reconstruction.

References

1. Nguyen, L. T., Schmidt, H. A., Von Haeseler, A. & Minh, B. Q. IQ-TREE: A fast and effective stochastic algorithm for estimating maximum-likelihood phylogenies. *Molecular Biology and Evolution* vol. 32 268–274 Article at https://doi.org/10.1093/molbev/msu300 (2015).
2. Goodstein, D. M. *et al.* Phytozome: A comparative platform for green plant genomics. *Nucleic Acids Res.* **40**, (2012).
3. Ryu, J. Y., Kim, H. U. & Lee, S. Y. Deep learning enables high-quality and high-throughput prediction of enzyme commission numbers. *Proc. Natl. Acad. Sci. U. S. A.* **116**, 13996–14001 (2019).
4. Swarbreck, D. *et al.* The Arabidopsis Information Resource (TAIR): Gene structure and function annotation. *Nucleic Acids Res.* **36**, 1009–1014 (2008).
5. Lamesch, P. *et al.* The Arabidopsis Information Resource (TAIR): Improved gene annotation and new tools. *Nucleic Acids Res.* **40**, D1202–D1210 (2012).
6. Armenteros, J. J. A. *et al.* Detecting sequence signals in targeting peptides using deep learning. *Life Sci. Alliance* **2**, (2019).
7. Emanuelsson, O., Nielsen, H., Brunak, S. & Von Heijne, G. Predicting subcellular localization of proteins based on their N-terminal amino acid sequence. *J. Mol. Biol.* **300**, 1005–1016 (2000).
8. Small, I., Peeters, N., Legeai, F. & Lurin, C. Predotar: A tool for rapidly screening proteomes for N-terminal targeting sequences. *Proteomics* **4**, 1581–1590 (2004).
9. Katoh, K. & Standley, D. M. MAFFT multiple sequence alignment software version 7: Improvements in performance and usability. *Molecular Biology and Evolution* vol. 30 772–780 Article at https://doi.org/10.1093/molbev/mst010 (2013).
10. Suyama, M., Torrents, D. & Bork, P. PAL2NAL: Robust conversion of protein sequence alignments into the corresponding codon alignments. *Nucleic Acids Res.* **34**, W609 (2006).
11. Gruber, A. V. & Feiz, L. Rubisco assembly in the chloroplast. *Front. Mol. Biosci.* **5**, (2018).

12. Chernomor, O. *et al.* Split diversity in constrained conservation prioritization using integer linear programming. *Methods Ecol. Evol.* **6**, 83–91 (2015).

Supplemental Figures

Figure S1

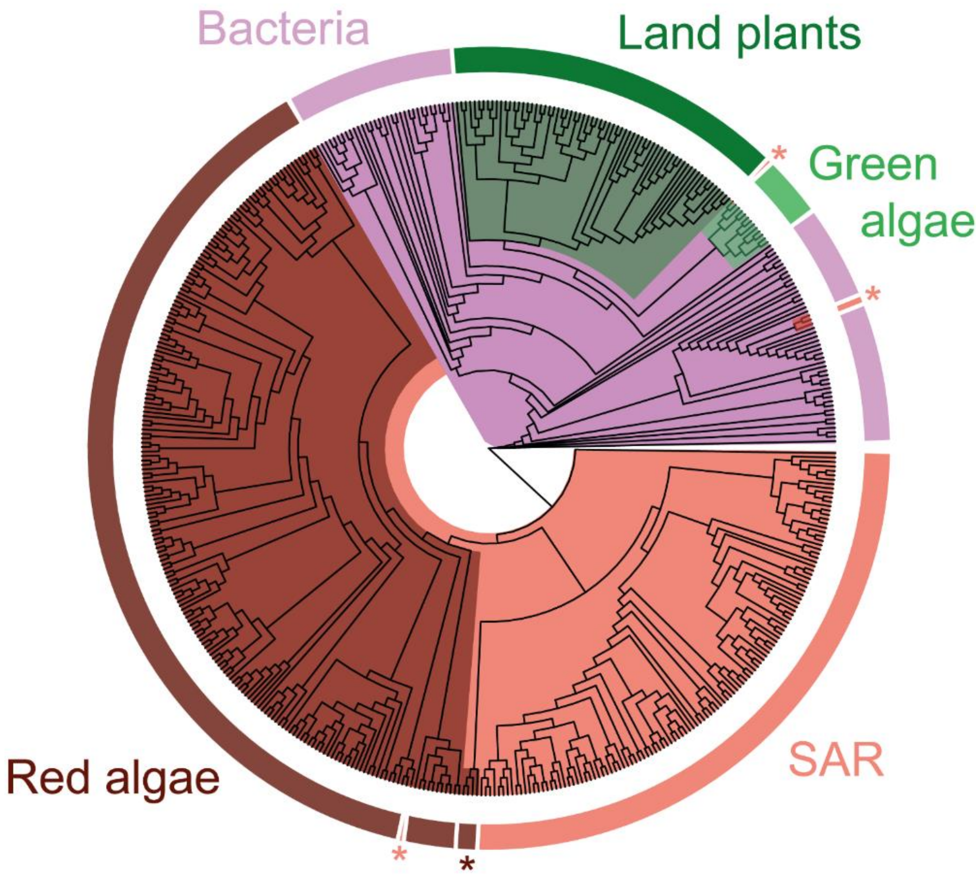

Figure S1. A phylogenetic tree of species in this study. Evolutionary history has been inferred from a multiple sequence alignment of the *rbcL* coding sequence in each species. The phylogeny is displayed as a cladogram for ease of visualisation, and species membership to different taxonomic groups (labelled) are highlighted by colour. Dark brown: red algae (*Rhodophyta*; *n* = 201). Light brown: SAR supergroup (*Stramenopiles*, *Alveolates*, and *Rhizaria*; *n* = 129). Lilac: bacteria (*Bacteria*; *n* = 78). Dark green: land plants (*Streptophyta*; *n* = 68). Light green: green algae (*Chlorophyta*; *n* = 12). Species at terminal nodes which cluster outside their designated taxonomic group are marked by an asterisk.

Figure S2

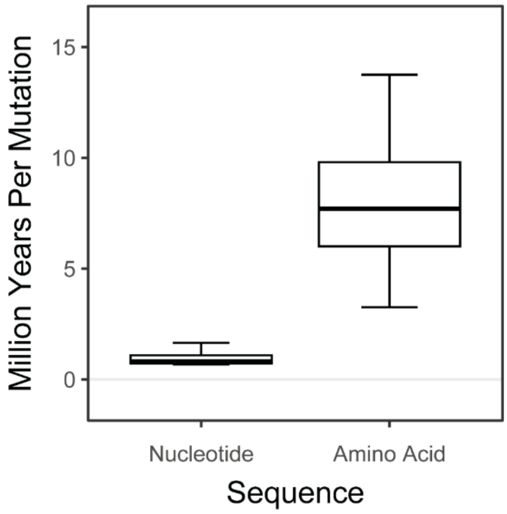

Figure S2. The rate of nucleotide and amino acid mutation in the rubisco large subunit experienced by C_3 angiosperms in the kinetic dataset, as expressed as the number of million years per individual sequence change. Molecular mutation rates were calculated since divergence from the last common ancestor at the base of the angiosperm clade 160 million years ago and considered an average *rbcL* of length 1428 nucleotide residues and 476 amino acid residues.

Figure S3

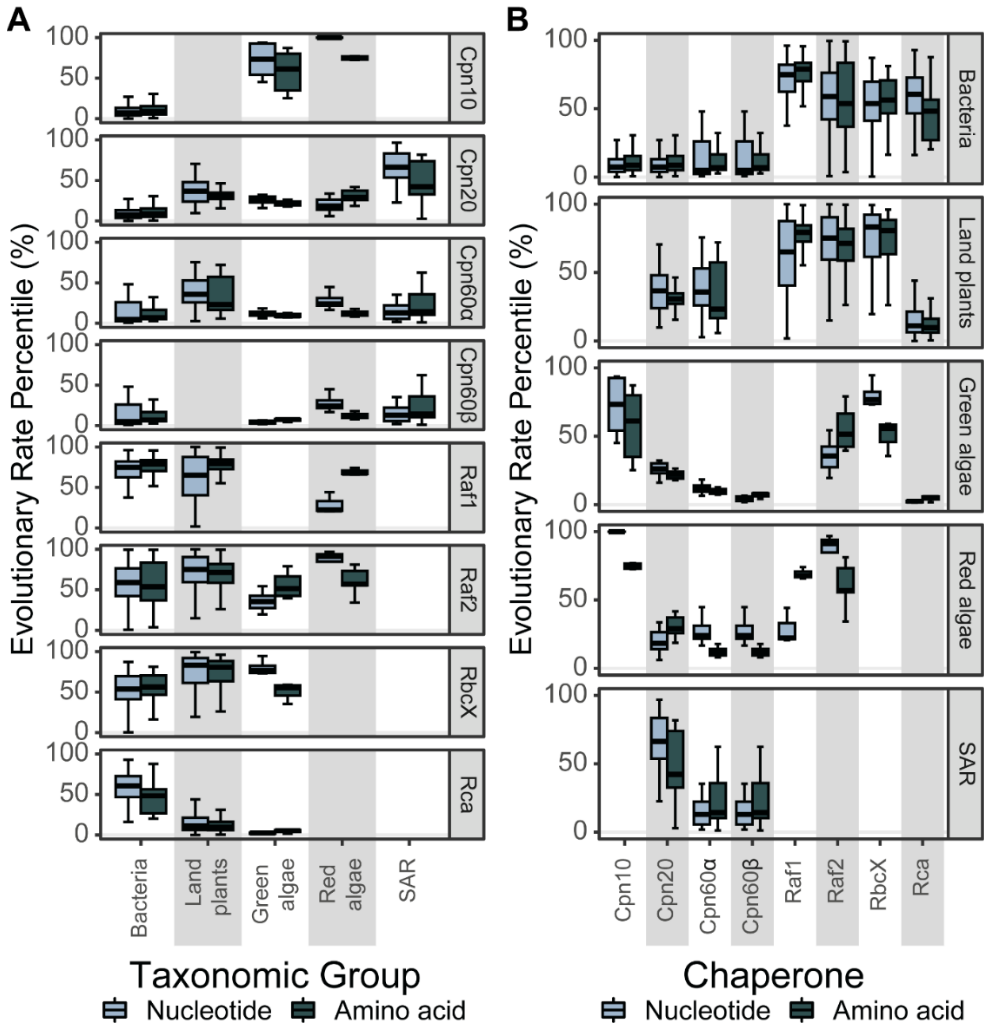

Figure S3. The extent of molecular evolution in each of rubisco's interacting chaperone partners. A) Boxplot of the extent of nucleotide and amino acid evolution (substitutions per sequence site) of each chaperone organised by taxonomic group. B) As in (A) but organised by gene/protein in alphabetical order. Chaperonin 60α: Cpn60α. Chaperonin 60β: Cpn60β. Chaperonin 10: Cpn10. Chaperonin 20: Cpn20. RbcX: RbcX. Rubisco accumulation factor 1: Raf1. Rubisco accumulation factor 2: Raf2. Rubisco activase: Rca. Although Bundle sheath defective 2 (Bsd2) is a known regulator of rubisco assembly, this chaperone was omitted from the analysis owing to insufficient sequence data. Data for this figure can be found in table S11.

Figure S4

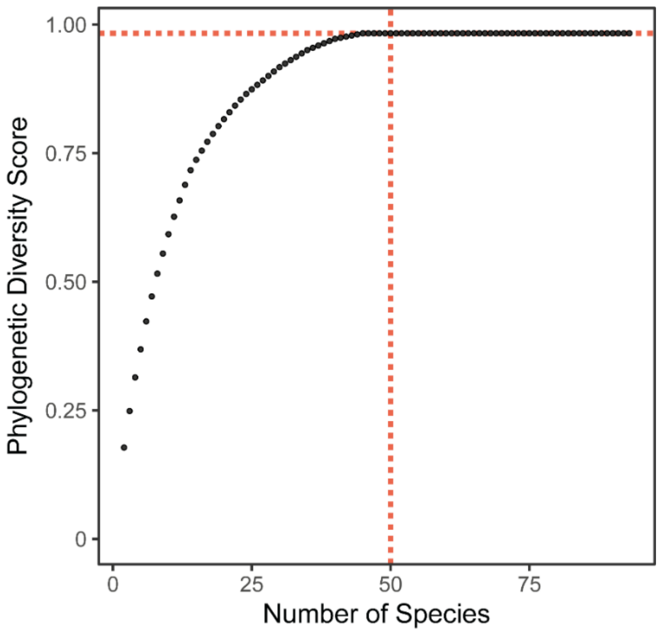

Figure S4. The maximal phylogenetic diversity score which can be obtained at each iterative sample size of species in the rubisco kinetic dataset (*n* = 2 – 93 species). Phylogenetic diversity is defined as the total tree length (i.e., the combined sum of all internal and terminal branch lengths) of the phylogenetic tree inferred for each optimal subset of species at each sample size. The sample size (*n* = 50) at which phylogenetic diversity plateaus is indicated by a red dashed line. The optimal species set at this threshold are shown in table S10.

Supplemental Tables

Table S1

Table S1. Summary of the sequence dataset used for analysis of rubisco molecular evolution in this study.

**Taxonomic Group**	***n***			
	**Species**	**Species Representative Gene Models**	***rbcL*/RbcL Sequences**	***rbcS*/RbcS Sequences**
Bacteria	78	32	78	86
Land plants	68	27	68	172
Green algae	12	4	12	24
Red algae	201	6	201	595
SAR	129	8	129	283
**Total**	**488**	**77**	**488**	**1140**

Table S2

Table S2. Summary of the percentile rate of nucleotide and protein evolution in each rubisco subunit in context of all other genes in each taxonomic group. The median, first quartile (Q1), third quartile (Q3), the interquartile range (IQR), the mean and the standard error (S.E.) are provided.

**Taxonomic Group**	**Rubisco Subunit**	**Sequence**	**Percentile Rate**					
			**Median**	**Q1**	**Q3**	**IQR**	**Mean**	**S.E.**
Bacteria	**RbcL**	**Nucleotide**	2.597	0.847	5.693	4.846	4.729	0.317
Land plants			0.057	0.016	0.162	0.146	0.357	0.100
Green algae			0.042	0.019	0.088	0.070	0.053	0.016
Red algae			0.043	0.030	0.192	0.162	0.132	0.040
SAR			0.575	0.269	2.005	1.736	1.653	0.527
Bacteria		**Protein**	2.652	0.952	5.129	4.177	3.993	0.246
Land plants			2.180	1.573	3.314	1.741	3.092	0.201
Green algae			0.983	0.478	1.703	1.224	1.178	0.344
Red algae			0.933	0.874	1.141	0.267	1.168	0.186
SAR			2.614	2.048	3.840	1.792	3.169	0.467
Bacteria	**RbcS**	**Nucleotide**	25.205	14.899	39.836	24.937	28.373	0.837
Land plants			64.057	52.487	76.510	24.023	61.857	1.089
Green algae			3.961	1.905	5.643	3.738	5.198	2.078
Red algae			1.256	1.010	1.920	0.910	1.381	0.201
SAR			5.185	1.106	10.724	9.618	7.636	1.532
Bacteria		**Protein**	41.927	24.678	52.779	28.101	39.030	0.842
Land plants			52.024	36.131	67.519	31.387	52.229	1.002
Green algae			14.338	10.363	17.657	7.293	14.623	2.020
Red algae			8.630	7.019	16.601	9.581	11.352	1.697
SAR			12.445	4.047	17.256	13.210	12.410	1.518

Table S3

Table S3. As in table S2 but in context of all other enzyme-encoding genes in each taxonomic group.

Taxonomic Group	Rubisco Subunit	Sequence	Percentile Rate					
			**Median**	**Q1**	**Q3**	**IQR**	**Mean**	**S.E.**
Bacteria	**RbcL**	**Nucleotide**	3.090	0.993	7.016	6.023	5.573	0.365
Land plants			0.070	0.031	0.174	0.143	0.384	0.108
Green algae			0.057	0.053	0.061	0.008	0.057	0.002
Red algae			0.093	0.077	0.161	0.085	0.152	0.037
SAR			0.498	0.241	1.316	1.075	1.275	0.452
Bacteria		**Protein**	2.978	0.961	5.706	4.745	4.334	0.274
Land plants			2.688	1.868	4.146	2.278	3.802	0.234
Green algae			0.229	0.192	0.286	0.094	0.334	0.115
Red algae			0.512	0.395	0.859	0.464	0.886	0.251
SAR			2.286	1.667	3.104	1.437	2.728	0.441
Bacteria	**RbcS**	**Nucleotide**	30.412	18.002	46.773	28.771	32.932	0.944
Land plants			71.256	60.765	83.175	22.410	68.478	1.055
Green algae			2.874	1.022	5.656	4.634	4.282	1.799
Red algae			1.044	0.525	2.056	1.531	1.382	0.299
SAR			6.122	0.819	11.510	10.692	8.097	1.639
Bacteria		**Protein**	47.314	29.298	56.569	27.271	43.035	0.887
Land plants			61.809	45.382	76.873	31.491	60.920	1.016
Green algae			17.982	12.037	23.533	11.496	17.801	2.583
Red algae			11.933	8.110	21.443	13.334	15.126	2.461
SAR			11.738	3.844	22.623	18.780	14.133	1.911

Table S4

Table S4. Summary of the rate of nucleotide and protein evolution in each Calvin-Benson-Bassham cycle enzyme as a percentage ratio (%) of that measured in the rubisco large subunit (*rbcL*/RbcL). The median, first quartile (Q1), third quartile (Q3), the interquartile range (IQR), the mean and the standard error (S.E.) are provided. RBCS: rubisco small subunit. PGK: phosphoglycerate kinase. GAPDH-A: glyceraldehyde-3-phosphate dehydrogenase A subunit. GAPDH-B: glyceraldehyde-3-phosphate dehydrogenase B subunit. TPI: triose phosphate isomerase. FBA: fructose-bisphosphate aldolase. FBP: fructose-1,6-bisphosphatase. TKL: transketolase. SBP: sedoheptulose-bisphosphatase. RPI: ribose 5-phosphate isomerase. RPE: ribulose-p-3-epimerase. PRK: phosphoribulokinase.

**Enzyme**	**Sequence**	**% of *rbcL*/RbcL Molecular Evolution**					
		**Median**	**Q1**	**Q3**	**IQR**	**Mean**	**S.E.**
RBCS	**Nucleotide**	336.230	303.768	465.258	161.490	14215.472	6977.094
PGK		194.398	154.133	260.690	106.557	10222.926	8653.225
GAPDH-A		211.457	181.128	268.329	87.201	12074.030	9018.351
GAPDH-B		146.379	112.891	298.844	185.954	3293.398	1821.487
TPI		216.343	173.907	284.861	110.954	5679.185	3315.866
FBA		221.158	168.605	302.040	133.435	10306.953	8831.565
FBP		292.943	235.488	399.049	163.561	10201.657	5517.012
TKL		239.675	202.167	303.532	101.365	9195.482	5239.744
SBP		232.694	204.043	305.004	100.962	4667.535	3302.289
RPI		426.887	268.135	694.738	426.602	15006.493	11123.542
RPE		204.113	181.546	283.966	102.419	5462.525	4008.166
PRK		223.418	159.157	288.776	129.619	3818.743	2612.882
RBCS	**Protein**	677.203	513.747	880.073	366.326	13630.350	6097.334
PGK		188.897	128.489	271.151	142.662	1478.183	1094.706
GAPDH-A		168.140	103.943	231.241	127.298	7539.044	5584.847
GAPDH-B		102.634	69.460	180.491	111.031	7341.694	5148.096

TPI	227.879	190.426	290.050	99.624	3948.902	2281.597
FBA	145.765	107.797	180.354	72.556	5257.912	5090.570
FBP	267.831	189.307	397.150	207.842	25149.320	19153.272
TKL	284.397	225.285	339.574	114.289	5429.319	3058.388
SBP	141.192	96.510	217.906	121.396	2784.952	2048.169
RPI	186.521	114.161	284.244	170.083	12075.717	11840.183
RPE	233.601	167.671	306.208	138.536	2686.190	1714.928
PRK	167.644	132.689	248.041	115.352	1892.755	1245.509

Table S5

Table S5. One-Sample Wilcoxon Signed Rank Test to assess significant differences in the rate of nucleotide and protein evolution between the rubisco large subunit (*rbcL*/RbcL) and each Calvin-Benson-Bassham cycle enzyme. A non-parametric test was used as data failed to conform to normality (Shapiro-Wilk test; $p < 0.05$). Statistics are rounded to three decimal places and corrected significance values are represented as α levels, where; $\alpha = 0.001$ if $P < 0.001$, $\alpha = 0.01$ if $0.001 < P < 0.01$, $\alpha = 0.05$ if $0.01 < P < 0.05$, and α = ns if $P > 0.05$. Calvin-Bensen-Bassham cycle enzymes/subunits are abbreviated following the convention in table S4.

**Enzyme**	**Nucleotide**		**Protein**	
	**Statistic**	***α***	**Statistic**	***α***
RBCS	2476425	0.001	2476417	0.001
PGK	51040	0.001	48937	0.001
GAPDH-A	55278	0.001	49267	0.001
GAPDH-B	2550	0.001	1689	0.01
TPI	59685	0.001	59570	0.001
FBA	8984	0.001	7727	0.001
FBP	60378	0.001	60261	0.001
TKL	60378	0.001	60378	0.001
SBP	59684	0.001	50188	0.001
RPI	37128	0.001	33896	0.001
RPE	45450	0.001	45030	0.001
PRK	50721	0.001	49118	0.001

Table S6

Table S6. Mean values of the ratio of rubisco large to small subunit percentile rank rate of evolution (*rbcL* to *rbcS* and RbcL to RbcS, respectively) and associated variation (±1 S.E.) in each taxonomic group.

**% Ratio**	**Taxonomic Group**				
	**Bacteria**	**Land Plants**	**Green Algae**	**Red Algae**	**SAR**
rbcL* : *rbcS	30.7 ± 4.3	0.6 ± 0.2	3.1 ± 1.6	10.7 ± 2.8	70.9 ± 46.8
**RbcL : RbcS**	12.2 ± 0.7	8.3 ± 1.2	8.3 ± 2.1	14.7 ± 3.0	40.2 ± 7.2

Table S7

Table S7. List of all species in each taxonomic group for which either a nuclear (land plants, green algae, red algae, SAR) or bacterial (bacteria) genome could be acquired.

Taxonomic Group	Species
**Bacteria**	Acaryochloris marina
	Acidithiobacillus ferrooxidans
	Allochromatium vinosum
	Anabaenopsis circularis
	Arthrospira platensis
	Aurantimonas manganoxydans
	Crocosphaera subtropica
	Gloeobacter kilaueensis
	Gloeobacter violaceus
	Gloeomargarita lithophora
	Halomicronema hongdechloris
	Hydrogenophaga pseudoflava
	Methylacidimicrobium cyclopophantes
	Methylacidimicrobium tartarophylax
	Methylacidiphilum fumariolicum
	Methylacidiphilum infernorum
	Microcystis aeruginosa
	Microcystis viridis
	Nocardia nova
	Nocardia seriolae
	Novimethylophilus kurashikiensis
	Phaeobacter gallaeciensis
	Phormidesmis priestleyi
	Planktothrix agardhii

	Prochlorococcus marinus
	Prochlorothrix hollandica
	Raphidiopsis brookii
	Synechococcus elongatus
	Thermosynechococcus elongatus
	Thermosynechococcus vulcanus
	Thioflexothrix psekupsii
	Trichormus variabilis
**Land Plants**	Aegilops tauschii
	Amaranthus hypochondriacus
	Arabidopsis thaliana
	Brassica napus
	Brassica oleracea
	Brassica rapa
	Camellia sinensis
	Capsicum annuum
	Cucumis sativus
	Dendrobium catenatum
	Glycine soja
	Gossypium hirsutum
	Hevea brasiliensis
	Hordeum vulgare
	Lactuca sativa
	Mucuna pruriens
	Nicotiana attenuata
	Oryza sativa
	Panicum virgatum
	Phaseolus vulgaris

	Salvia splendens
	Sorghum bicolor
	Spinacia oleracea
	Triticum aestivum
	Triticum turgidum
	Triticum urartu
	Zea mays
**Green Algae**	Botryococcus braunii
	Chromochloris zofingiensis
	Dunaliella salina
	Volvox carteri
**Red Algae**	Chondrus crispus
	Cyanidiococcus yangmingshanensis
	Galdieria sulphuraria
	Gracilariopsis chorda
	Porphyra umbilicalis
	Porphyridium purpureum
**SAR**	Aureococcus anophagefferens
	Ectocarpus siliculosus
	Fistulifera solaris
	Microchloropsis salina
	Nannochloropsis gaditana
	Phaeodactylum tricornutum
	Thalassiosira oceanica
	Thalassiosira pseudonana

Table S8

Table S8. The gene loci encoding the photosynthetic isoforms of Calvin-Benson-Bassham cycle enzymes in *Arabidopsis thaliana*.

Enzyme	Unique ID	Gene name	Arabidopsis TAIR ID
PGK	PGK1	PHOSPHOGLYCERATE KINASE 1	AT3G12780
	PGK2	PHOSPHOGLYCERATE KINASE 2	AT1G56190
GAPDH-A	GAPA-1	GLYCERALDEHYDE 3-PHOSPHATE DEHYDROGENASE A SUBUNIT 1	AT3G26650
	GAPA-2	GLYCERALDEHYDE 3-PHOSPHATE DEHYDROGENASE A SUBUNIT 2	AT1G12900
GAPDH-B	GAPB	GLYCERALDEHYDE-3-PHOSPHATE DEHYDROGENASE B SUBUNIT	AT1G42970
TPI	TPI	TRIOSEPHOSPHATE ISOMERASE	AT2G21170
FBA	FBA1	FRUCTOSE-BISPHOSPHATE ALDOLASE 1	AT2G21330
	FBA2	FRUCTOSE-BISPHOSPHATE ALDOLASE 2	AT4G38970
FBP	FBP1	FRUCTOSE 1,6-BISPHOSPHATE PHOSPHATASE	AT3G54050
TKL	TKL1	TRANSKETOLASE 1	AT3G60750
SBP	SBP	SEDOHEPTULOSE-BISPHOSPHATAS	AT3G55800
RPI	RPI	RIBOSE 5-PHOSPHATE ISOMERASE	AT3G04790
RPE	RPE	D-RIBULOSE-5-PHOSPHATE-3-EPIMERASE	AT5G61410
PRK	PRK	PHOSPHORIBULOKINASE	AT1G32060

Table S9

Table S9. The gene loci encoding the chaperones involved in Form I rubisco assembly and metabolic regulation in *Arabidopsis thaliana*.

Chaperone	Unique ID	Gene name	Arabidopsis TAIR ID
Bsd2	Bsd2	BUNDLE SHEATH DEFECTIVE 2	AT3G47650
Cpn60α	Cpn60α_1	CHAPERONIN-60ALPHA1	AT2G28000
	Cpn60α_2	CHAPERONIN-60ALPHA2	AT5G18820
Cpn60β	Cpn60β_1	CHAPERONIN-60BETA1	AT1G55490
	Cpn60β_2	CHAPERONIN-60BETA2	AT3G13470
	Cpn60β_3	CHAPERONIN-60BETA3	AT5G56500
	Cpn60β_4	CHAPERONIN-60BETA4	AT1G26230
Cpn10	Cpn10_1	CHLOROPLAST CHAPERONIN 10	AT2G44650
	Cpn10_2	GROES	AT3G60210
Cpn20	Cpn20	CHAPERONIN 20	AT5G20720
RbcX	RbcX_1	RBCX1	AT4G04330
	RbcX_2	RBCX2	AT5G19855
Raf1	Raf1_1	RUBISCO ACCUMULATION FACTOR-LIKE PROTEIN	AT5G28500
	Raf1_2	RUBISCO ACCUMULATION FACTOR 1	AT3G04550
Raf2	Raf2	RUBISCO ASSEMBLY FACTOR 2	AT5G51110
Rca	Rca	RUBISCO ACTIVASE	AT2G39730

Table S10

Table S10. The subset of 50 C_3 angiosperms which captured the vast majority of phylogenetic diversity encapsulated in the full rubisco kinetic dataset, as used for the basis of the analysis in table S12.

**Division**	**Species**
**Dicot**	Agriophyllum squarrosum
	Amphicarpaea bracteata
	Artemisia myriantha
	Artemisia vulgaris
	Beta vulgaris
	Chenopodiastrum murale
	Chenopodium album
	Citrullus ecirrhosus
	Desmodium cinereum
	Desmodium intortum
	Erythrina flabelliformis
	Euphorbia helioscopia
	Euphorbia microsphaera
	Flaveria cronquistii
	Flaveria pringlei
	Flueggea suffruticosa
	Foeniculum vulgare
	Glycine canescens
	Lepidium campestre
	Macrotyloma uniflorum
	Manihot esculenta
	Mercurialis annua
	Nicotiana tabacum

	Oxybasis rubra
	Phaseolus coccineus
	Pueraria montana
	Sphenostylis stenocarpa
	Spinacia oleracea
	Tephrosia candida
	Tephrosia purpurea
	Tephrosia rhodesica
**Monoocot**	Aegilops comosa
	Aegilops speltoides
	Agrostis stolonifera
	Arctagrostis latifolia
	Brachypodium distachyon
	Bromus anomalus
	Calamagrostis stricta subsp. inexpansa
	Deschampsia danthonioides
	Elymus farctus
	Lolium giganteum
	Lolium multiflorum
	Musa velutina
	Oryza barthii x Oryza glaberrima
	Oryza eichingeri
	Oryza glaberrima
	Panicum milioides
	Poa palustris
	Puccinellia distans
	Puccinellia maritima

Table S11

Table S11. Summary of the percentile rate of nucleotide and protein evolution in each of rubisco's interacting chaperone partners in context of all other genes in each taxonomic group. Data have been calculated between each pairwise combination of species within a taxonomic group for which either a nuclear (land plants, green algae, red algae, SAR) or bacterial (bacteria) genome could be acquired. Statistics are rounded to three decimal places, and include the median, first quartile (Q1), third quartile (Q3), the interquartile range (IQR), the mean and the standard error (S.E.). Rows and column containing N/A values denote missing data (see Methods).

Taxonomic Group	Rubisco Subunit	Sequence	Percentile Rate					
			Median	Q1	Q3	IQR	Mean	S.E.
Bacteria	**Rca**	**Nucleotide**	60.600	46.645	72.651	26.006	62.542	2.860
Land plants			11.058	6.231	21.374	15.142	17.656	1.000
Green algae			2.531	1.746	2.833	1.087	3.023	0.854
Red algae			N/A	N/A	N/A	N/A	N/A	N/A
SAR			N/A	N/A	N/A	N/A	N/A	N/A
Bacteria		**Protein**	48.212	26.908	56.477	29.569	48.414	3.275
Land plants			9.685	5.996	16.183	10.187	15.384	0.921
Green algae			5.226	3.735	6.019	2.284	5.330	1.213
Red algae			N/A	N/A	N/A	N/A	N/A	N/A
SAR			N/A	N/A	N/A	N/A	N/A	N/A
Bacteria	**Cpn10**	**Nucleotide**	7.383	3.834	13.185	9.352	10.065	0.410
Land plants			N/A	N/A	N/A	N/A	N/A	N/A
Green algae			73.334	54.076	92.558	38.482	72.007	9.232
Red algae			99.916	99.895	99.927	0.032	99.906	0.017
SAR			N/A	N/A	N/A	N/A	N/A	N/A
Bacteria		**Protein**	8.805	5.116	15.393	10.277	10.959	0.341
Land plants			N/A	N/A	N/A	N/A	N/A	N/A
Green algae			61.114	34.853	79.986	45.133	57.826	11.191

Red algae			74.744	72.862	76.651	3.789	74.769	1.162
SAR			N/A	N/A	N/A	N/A	N/A	N/A
Bacteria	**Cpn20**	**Nucleotide**	7.383	3.834	13.185	9.352	10.065	0.410
Land plants			36.601	23.947	47.920	23.973	37.041	0.791
Green algae			26.103	22.948	30.081	7.133	25.633	2.448
Red algae			18.322	14.026	26.449	12.423	20.410	2.235
SAR			66.293	53.703	83.382	29.678	66.736	3.769
Bacteria		**Protein**	8.805	5.116	15.393	10.277	10.959	0.341
Land plants			30.637	27.030	34.859	7.828	31.244	0.402
Green algae			22.078	19.036	23.944	4.908	21.814	1.401
Red algae			29.005	25.746	37.267	11.521	31.175	1.994
SAR			42.253	32.649	73.838	41.189	49.615	4.107
Bacteria	**Cpn60α**	**Nucleotide**	4.971	2.428	26.083	23.655	13.896	1.686
Land plants			35.767	25.918	53.009	27.091	39.147	1.066
Green algae			11.153	9.382	14.049	4.667	11.803	1.736
Red algae			24.023	21.721	31.270	9.549	27.034	2.148
SAR			12.939	5.571	22.243	16.672	16.451	2.728
Bacteria		**Protein**	6.870	5.314	16.609	11.294	11.737	0.987
Land plants			23.233	16.681	57.310	40.628	31.709	1.151
Green algae			9.104	8.050	11.442	3.392	9.664	0.917
Red algae			12.200	9.516	14.251	4.735	12.153	0.758
SAR			14.277	10.334	35.866	25.532	22.250	2.899
Bacteria	**Cpn60β**	**Nucleotide**	4.971	2.428	26.083	23.655	13.896	1.686
Land plants			N/A	N/A	N/A	N/A	N/A	N/A
Green algae			4.213	2.687	6.059	3.372	4.254	0.841
Red algae			24.023	21.721	31.270	9.549	27.034	2.148
SAR			12.939	5.571	22.243	16.672	16.451	2.728
Bacteria		**Protein**	6.870	5.314	16.609	11.294	11.737	0.987

Land plants			N/A	N/A	N/A	N/A	N/A	N/A
Green algae			7.646	6.150	8.271	2.121	7.614	1.105
Red algae			12.200	9.516	14.251	4.735	12.153	0.758
SAR			14.277	10.334	35.866	25.532	22.250	2.899
Bacteria	**Raf1**	**Nucleotide**	74.861	62.447	82.097	19.650	71.816	1.259
Land plants			65.047	40.394	87.520	47.126	64.306	1.594
Green algae			N/A	N/A	N/A	N/A	N/A	N/A
Red algae			22.207	21.367	33.185	11.818	28.966	7.614
SAR			N/A	N/A	N/A	N/A	N/A	N/A
Bacteria		**Protein**	78.876	70.092	83.569	13.477	76.585	1.079
Land plants			79.334	72.528	84.471	11.944	76.991	0.898
Green algae			N/A	N/A	N/A	N/A	N/A	N/A
Red algae			67.652	66.648	70.873	4.225	69.130	2.549
SAR			N/A	N/A	N/A	N/A	N/A	N/A
Bacteria	**Raf2**	**Nucleotide**	58.842	42.203	76.289	34.086	56.800	1.781
Land plants			75.092	59.362	90.363	31.002	73.069	1.087
Green algae			35.517	27.328	42.532	15.205	35.756	5.167
Red algae			91.233	84.730	93.894	9.163	83.658	8.248
SAR			N/A	N/A	N/A	N/A	N/A	N/A
Bacteria		**Protein**	53.812	36.805	83.573	46.768	56.263	1.887
Land plants			71.317	58.666	81.904	23.238	70.344	0.957
Green algae			51.523	42.443	66.557	24.114	55.425	6.637
Red algae			56.872	55.599	73.289	17.691	60.219	8.129
SAR			N/A	N/A	N/A	N/A	N/A	N/A
Bacteria	**RbcX**	**Nucleotide**	53.789	41.228	69.727	28.499	54.376	1.488
Land plants			83.203	61.384	92.168	30.784	74.490	1.323
Green algae			76.765	73.652	82.446	8.795	77.523	4.688
Red algae			N/A	N/A	N/A	N/A	N/A	N/A

SAR		N/A	N/A	N/A	N/A	N/A	N/A
Bacteria	**Protein**	56.356	46.610	70.736	24.125	56.760	1.243
Land plants		80.612	63.306	88.452	25.146	74.871	0.968
Green algae		55.560	45.803	58.422	12.619	55.154	6.568
Red algae		N/A	N/A	N/A	N/A	N/A	N/A
SAR		N/A	N/A	N/A	N/A	N/A	N/A

Table S12

Table S12. The pairwise correlation coefficient (% variation explained), associated significance and direction of association from the results of the linear regression analysis between the extent of RbcL protein evolution and each rubisco kinetic trait but when considering the minimum combination of species which optimize phylogenetic diversity of species in the kinetic dataset, as predicated on Figure S4 and table S10. Significance values are represented as α levels, where α = 0.001 if P < 0.001, α = 0.01 if 0.001 < P < 0.01, α = 0.05 if 0.01 < P < 0.05, and α = ns if P > 0.05.

Kinetic Trait	% Var Explained	*α*	Direction
$S_{C/O}$	13.5	0.01	+
k_{catC}	7.5	0.05	+
K_{catC}/k_{C}	6.4	0.05	+
K_{C}	ns	ns	ns
K_{C}^{air}	ns	ns	ns
K_{O}	ns	ns	ns
K_{C}/K_{O}	ns	ns	ns

Table S13

Table S13. The pairwise correlation coefficient (% variation explained), associated significance and direction of association from the results of the linear regression analysis between the extent of RbcL protein evolution and each rubisco kinetic trait, but from the analysis of independent phylogenetic trees inferred from the same set of species sequence data but using alternative models of sequence evolution. Significance values are represented as α levels, where $\alpha = 0.001$ if $P < 0.001$, $\alpha = 0.01$ if $0.001 < P < 0.01$, $\alpha = 0.05$ if $0.01 < P < 0.05$, and α = ns if $P > 0.05$.

Tree Model	Kinetic Trait	% Var Explained	*α*	Direction
**LG+I+G4**	$S_{C/O}$	7.2	0.01	+
	k_{catC}	11.0	0.001	+
	K_{catC}/k_C	9.6	0.01	+
	K_C	ns	ns	ns
	K_C^{air}	ns	ns	ns
	K_O	ns	ns	ns
	K_C/K_O	ns	ns	ns
**JTTDCMut+I+G4**	$S_{C/O}$	7.5	0.01	+
	k_{catC}	5.1	0.05	+
	K_{catC}/k_C	12.3	0.001	+
	K_C	ns	ns	ns
	K_C^{air}	ns	ns	ns
	K_O	ns	ns	ns
	K_C/K_O	ns	ns	ns
